# Bilateral injection-site granuloma by subcutaneous administration of luteinizing hormone-releasing hormone analogue: a case report

**DOI:** 10.4076/1757-1626-2-8326

**Published:** 2009-09-15

**Authors:** Riko Kitazawa, Fukashi Yamamichi, Toshiharu Hidaka, Shinichi Morishita, Takeshi Kondo, Kiyoshi Mori, Sohei Kitazawa

**Affiliations:** 1 Division of Pathology, Department of Pathology, Kobe University Graduate School of Medicine7-5-1 Kusunoki-cyo, Chuo-ku, Kobe 650-0017Japan; 2 Division of Urology, Kobe 100 Years Memorial Hospital1-9-1 Misaki-cho, Hyogo-ku, Kobe 652-0855Japan; 3 Division of Surgery, Kobe 100 Years Memorial Hospital1-9-1 Misaki-cho, Hyogo-ku, Kobe 652-0855Japan

## Abstract

We report a typical case of injection-site granuloma attributed to subcutaneous administration of leuprorelin acetate, an LHRH agonist. A 70-year-old man who had undergone total prostatectomy and was subsequently given leuprorelin injections for prostatic cancer presented with bilateral nodules in the lower abdominal wall. An excisional biopsy revealed a non-caseous epithelioid granuloma consisting of CD-68 positive histiocytic cells with infiltration of T-lymphocytes and eosinophils; skin metastasis from prostatic adenocarcinoma was ruled out through histological and immunohistochemical analysis. Generally, granulomas may be caused by delayed-type hypersensitivity to the constituents of leuprorelin acetate injections.

## Introduction

Although clinically localized prostatic cancer is treated by prostatectomy or radiotherapy (external beam radiotherapy or brachytherapy), endocrine therapy is chosen for advanced or metastatic cancer because most prostatic cancer cells depend on androgens for their growth. To deprive of androgens, synthetic agonists of luteinizing hormone-releasing hormone (LHRH) are administrated instead of conducting an orchiectomy [1]. LHRH agonists, after the initial transient flare-up of LH secretion, suppress LH release and sex hormones by down-regulating the receptors of gonadotrophs of the pituitary gland and ablating androgens. Although decrease of libido and osteopenia are well-known as the major side effects of LHRH agonists, we describe a rare case of injection-site granuloma (attributed to subcutaneous administration of an LHRH agonist) and emphasize its importance since it could mimic cancer metastasis, as in this case.

## Case presentation

A 70-year-old Japanese man presenting with bilateral subcutaneous nodules in the lower abdominal region was referred to our hospital. He had undergone total prostatectomy 6 months earlier and had then received monthly subcutaneous injections of 3.7 mg leuprorelin acetate, a synthetic LHRH agonist. The bilateral nodules in the lower abdomen were located at the sites of the leuprorelin injections. The nodules were excised to rule out skin metastasis from prostatic cancer.

### Pathologic findings

**Figure 1.**
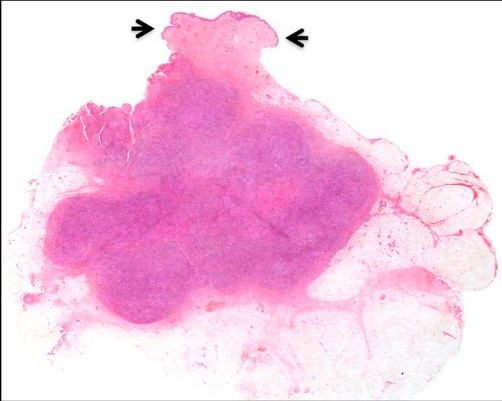
Cross-section of the lesion excised from the right lower abdomen. Epidermis covering the lesion (arrows) was intact. The well-circumscribed nodules, 17 × 15 mm, were located in the upper dermis to subcutaneous adipose tissue (HE, X10).

**Figure 2.**
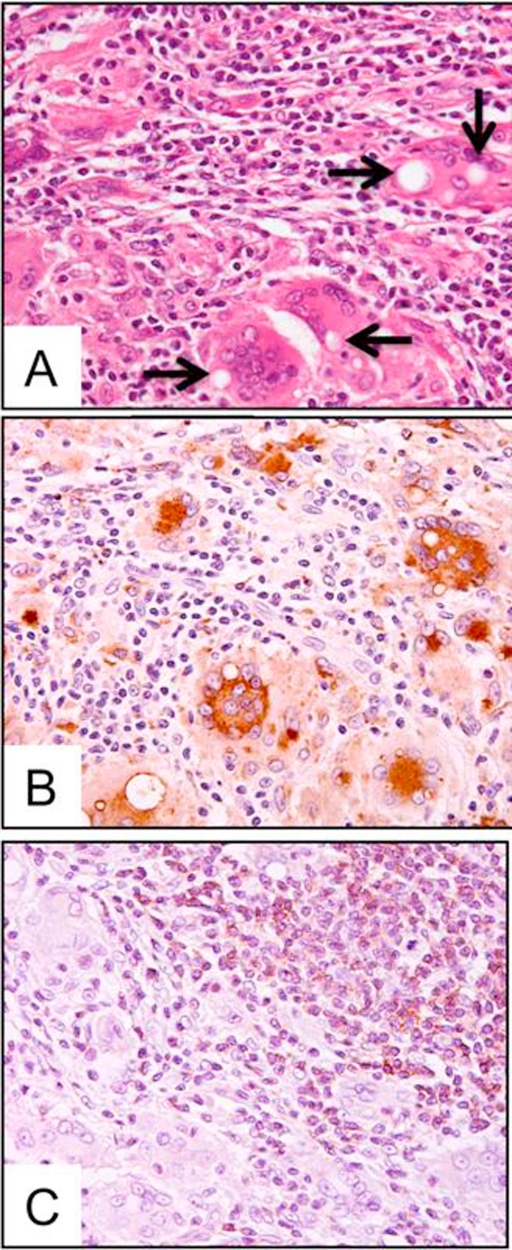
Microscopic findings of non-caseous epithelioid granulomatous lesion. **(A):** High-power view of the nodule revealed epithelioid cells and multinucleated giant cells with droplets (arrows) and infiltration of eosinophils and lymphocytes (HE, X400). **(B):** Immunohistochemical staining for CD-68. Epithelioid cells and multi-nucleated giant cells were positive for CD-68. **(C):** Immunohistochemical staining for CD-3. (counterstained with Hematoxylin, X400).

Grossly, the well-circumscribed nodules, approximately 20 mm in diameter, were located in the upper dermis of subcutaneous adipose tissue (Figure 1). Microscopically, they were non-caseous granulomatous lesions with multi-nucleated giant cells containing lipid droplets infiltrated with lymphocytes and eosinophils (Figure 2A). Ziehl-Neelsen, Periodic acid-Schiff (PAS) and Grocott staining showed no bacterial, fungal or protozoan component. Immunohistochemically, epithelioid cells were negative for cytokeratin 7, cytokeratin 20, low molecular weight keratin (CAM5.2) and prostate specific antigen (PSA), indicating that metastasis from prostatic cancer could be ruled out. Furthermore, epithelioid cells and multi-nucleated giant cells positive for CD68 (Fig. 2B) were histiocytic cells; infiltrating lymphocytes positive for CD-3 (Figure 2C), but negative for CD-20, were T-lymphocytes. These findings suggested that the granulomas at the injection sites were caused by delayed-type hypersensitivity to the leuprorelin acetate.

## Discussion

Long-acting, synthetic LHRH agonists such as leuprorelin and goserelin acetate have been widely used for advanced prostatic cancer [2]. Major side effects of LHRH analogues are hot flashes, osteopenia and decreased libido due to androgen deprivation. Injection-site granuloma is associated with insulin and aluminum-containing tetanus toxoid vaccines [3]. Injections of LHRH analogue rarely cause skin lesion-mimicking metastasis of prostatic cancer [4-6]. In this case, histological and immunohistochemical analysis of the lesions at the injection sites ruled out skin metastasis and infection by microorganisms. Granulomas consisting of CD-68 positive histiocytes and CD3-positive T lymphocytes were compatible with those seen in delayed-type hypersensitivity reactions; droplet-containing phagosomes were also observed in mono- and multi-nucleated histiocytes. Since leuprorelin acetate is combined with lactic acid, glycolic acid co-polymers or lactic acid polymers, the formation of granulomas may be related to the co-polymers or to the LHRH analogue itself. Indeed, Manasco *et al.* have demonstrated that the polymer, and not the diluent of leuprorelin acetate, elicits a skin reaction, concluding that the causative agent is the polymer [7]. Therefore, in prostatic cancer patients treated with LHRH analogues, both the remission due to the expansion of testosterone-insensitive clones and the possible failure of leuprorelin administration because of local skin reaction should be taken into consideration. In summary, we reported a case of bilateral injection-site granuloma by leuprorelin administration.
